# Follicular Helper T Cells in Systemic Lupus Erythematosus: Why Should They Be Considered as Interesting Therapeutic Targets?

**DOI:** 10.1155/2016/5767106

**Published:** 2016-08-22

**Authors:** Matthieu Sawaf, Hélène Dumortier, Fanny Monneaux

**Affiliations:** CNRS, Immunopathologie et Chimie Thérapeutique/Laboratory of Excellence MEDALIS, Institut de Biologie Moléculaire et Cellulaire, 67084 Strasbourg, France

## Abstract

Systemic lupus erythematosus (SLE) is a chronic autoimmune disease characterized by B cell hyperactivity leading to the production of autoantibodies, some of which having a deleterious effect. Reducing autoantibody production thus represents a way of controlling lupus pathogenesis, and a better understanding of the molecular and cellular factors involved in the differentiation of B cells into plasma cells could allow identifying new therapeutic targets. Follicular helper T cells (T_FH_) represent a distinct subset of CD4^+^ T cells specialized in providing help to B cells. They are required for the formation of germinal centers and the generation of long-lived serological memory and, as such, are suspected to play a central role in SLE. Recent advances in the field of T_FH_ biology have allowed the identification of important molecular factors involved in T_FH_ differentiation, regulation, and function. Interestingly, some of these T_FH_-related molecules have been described to be dysregulated in lupus patients. In the present review, we give an overview of the aberrant expression and/or function of such key players in lupus, and we highlight their potential as therapeutic targets.

## 1. Introduction

Systemic lupus erythematosus (SLE) is a severe systemic autoimmune disease and, as such, is characterized by a loss of self-tolerance. The etiology of SLE is not well defined, but genetic, hormonal, and environmental factors, as well as immune disorders, are likely implicated. During SLE, inflammation leads to damage of various tissues, including the joints, skin, kidneys, heart, lungs, blood vessels, and brain. Dysregulation of various components of the immune system can be observed at different stages of disease development, but hyperactivity of B cells, leading to excessive production of multiple autoantibodies (autoAb), is one of the major immunological stigmata of SLE. Indeed, SLE is characterized by the production of antinuclear autoAb (e.g., autoAb specific for chromatin) and by the formation of immune complexes, which contribute to tissue damage. Deposits of immune complexes in organs such as kidneys lead to subsequent inflammation through the activation of the complement system and the recruitment of inflammatory cells. The presence of autoAb is an absolute prerequisite for the development of lupus nephritis [1] and, interestingly, we demonstrated that pathogenic autoAb can be locally produced by plasma cells, which have homed to inflamed kidneys of lupus mice [2]. B cells and derivatives (plasma cells) are thus considered at the center of SLE pathogenesis and this is supported by the observation of a high frequency of plasma cell precursors in the blood of children with SLE [3]. Furthermore, an increase of circulating plasma cells in lupus patients is correlated with disease activity [4].

The generation of Ab can occur via the extrafollicular or the germinal center (GC) responses. The extrafollicular response leads to short-lived plasma cells, which do not go through the affinity maturation process. In contrast, the GC is the theater of intense cell collaboration between GC B cells and follicular helper T cells (T_FH_) leading to the differentiation of long-lived plasma cells harboring high antigen-specificity. Interestingly, lupus autoAb are high affinity, somatically mutated, and class-switched immunoglobulin (Ig)G [5] indicating T and B cell collaboration [6] and intense GC activity. Therefore, it is likely that a dysfunction in B cell differentiation mechanisms occurs in lupus, leading to excessive numbers of autoreactive plasma cells. It is particularly attracting and plausible to envisage that a dysregulation of T_FH_ could be the underlying key factor.

In this review, we succinctly expose recent understanding in T_FH_ biology (described in detail elsewhere; see [7] for review), in order to introduce important molecular factors involved in T_FH_ differentiation, regulation, and function. We then give an overview of the aberrant expression and/or function of such key players in lupus patients, and we highlight their potential as therapeutic targets.

## 2. T_FH_ Cells: From Their Generation to Their Regulation

The generation of high affinity Ab requires T/B interactions that mainly occur in GC. T_FH_ cells represent a distinct subset of CD4^+^ T cells involved in GC formation and specialized in providing help to B cells to differentiate into plasma cells or memory B cells [8]. T_FH_ express high levels of CXC chemokine receptor type 5 (CXCR5), PD-1 (Programmed Death-1), ICOS (Inducible T cell CO-Stimulator), and the regulator transcription factor Bcl6 (B cell lymphoma 6), which provide excellent markers for their identification. Moreover, secretion of high levels of IL-21 is a critical characteristic of T_FH_ cells.

**Figure 1:**
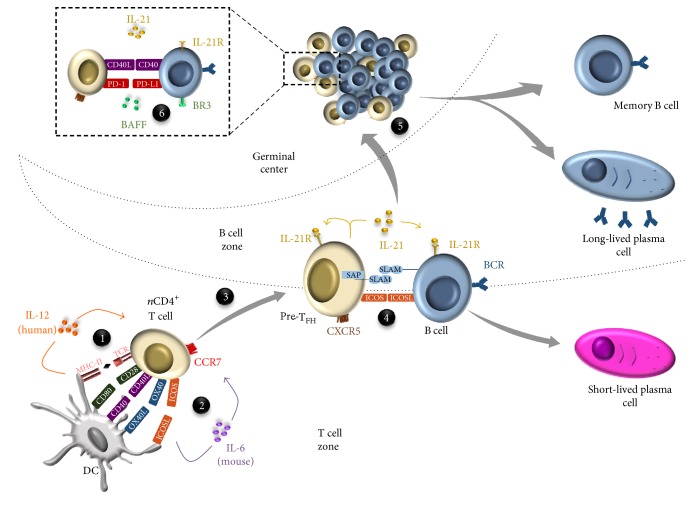
T_FH_ differentiation in secondary lymphoid organs is a multistep process required to establish a high affinity antibody response. (1) Naive CD4^+^ T cells localized in the T cell zone are first primed by DC thanks to MHCII-peptide-TCR interactions. (2) Once activated, CD4^+^ T cells upregulate costimulatory molecules such as CD40L, OX40, and ICOS, favoring their crosstalk with DC. Combined with this interaction DC-derived cytokines (IL-6 in mice and IL-12 in humans) drive differentiation of activated T cell into pre-T_FH_ cells. (3) Thanks to CXCR5 upregulation and CCR7 downregulation, pre-T_FH_ cells are attracted to the T-B border by a CXCL13 gradient. (4) A SAP/SLAM-stabilized interaction between ICOSL-expressing B cells and pre-T_FH_ cells occurs at the T-B border, finalizing T_FH_ cell differentiation. (5) Finally, mature T_FH_ cells migrate toward the GC, where they provide help to B cells. This crosstalk induces both B cell differentiation in plasma cells and memory B cells, thanks to IL-21/IL-21R and CD40/CD40L signals, and B cell survival via BAFF/BR3 and PD1/PD-L1 interactions (6).

T_FH_ are generated after immunization or infection following the interaction of naive CD4^+^ T cells with dendritic cells (DC) within the T cell zone of secondary lymphoid organs (SLO). Signals provided by DC induce the expression of a myriad of proteins (transcription factors, surface molecules, and cytokines) that are essential for T_FH_ generation, migration, and function. In fact, T_FH_ differentiation is a multistage process (Figure 1), which can be sequentially defined as follows: (i) naive CD4^+^ T cells are activated by DC (thanks to the MHC-peptide complex/TCR interaction) in the T cell zone and become immature T_FH_ (also called pre-T_FH_) [9]; (ii) newly generated pre-T_FH_ then migrate to the interfollicular zone, where cognate interactions with B cells allow the final maturation step; (iii) these mature T_FH_ reach the GC in which T_FH_-GC B cell interactions will favor isotype class switch, somatic hypermutations, and affinity maturation.

### 2.1. Pre-T_FH_ Generation: DC as the Stage Director

The initial priming of CD4^+^ T cells requires cognate interactions and costimulatory signals delivered by DC through CD40, CD80/86, ICOSL, and OX40L (Table 1). CD28 (that binds CD80/86) was shown to be essential to T_FH_ development as mice deficient for CD28 display CD4^+^ T cells that fail to upregulate CXCR5 and OX40, leading to disrupted GC formation [10]. In addition, upregulation of OX40L on DC following CD40-induced maturation allows CXCR5 expression by OX40^+^ T cells [11]. Moreover, ICOS signaling leads to an increased expression of the transcription factors Bcl6 and Ascl2 (achaete-scute homologue-2). The latter promote both the reciprocal CXCR5 upregulation and CCR7 downregulation on activated CD4^+^ T cells, which then become pre-T_FH_ [12, 13]. In turn, Bcl6 induces the expression of ICOS, PD1, CD40L, and SAP (SLAM- (Signaling Lymphocytic Activation Molecule-) Associated Protein; critical for T-B interaction).

Cytokines secreted by DC also play a pivotal role in pre-T_FH_ development (Table 1). IL-6, a DC-derived proinflammatory cytokine, has been demonstrated to be the main soluble factor driving T_FH_ differentiation in mice [14]. In humans, IL-12 has been shown to be the key cytokine that promotes T_FH_-like cell differentiation [15, 16]. If, in the initial work, neither IL-6 nor IL-21 were described as being able to promote T_FH_ differentiation [15], a recent study suggests that human plasmablasts produce IL-6, which is responsible for the subsequent differentiation of naive CD4^+^ T cells into B cell helpers CXCR5^+^ICOS^+^Bcl6^+^IL-21^+^ T cells [17]. IL-21 is required for T_FH_ function but it is also an important factor for T_FH_ generation [18] and, interestingly, both IL-6 and IL-12 are potent inducers of IL-21 expression in mice [19] and humans, respectively [15]. As IL-21 is an autocrine cytokine for pre-T_FH_ generation, further studies are required to better clarify individual cytokine contributions. Cytokine signaling involves the subsequent activation of Janus kinase-STAT (Signal Transducer and Activator of Transcription) signaling pathway. STAT3 is a major signaling molecule for IL-6 and IL-21 [20, 21], whereas IL-12 signaling occurs through STAT4 activation. However, IL-12-induced expression of IL-21 by human CD4^+^ T cells is compromised in patients with functional STAT3 deficiency, suggesting that IL-12 ability to promote IL-21-producing CD4^+^ T cells is predominantly STAT3 dependent [22]. Moreover, STAT3-deficient patients have reduced numbers of circulating T_FH_-like cells [23]. Altogether, these data suggest that the STAT3 signaling pathway plays an important role in T_FH_ differentiation and subsequent B cell help.

During this first step of the T_FH_ differentiation process, both cell surface interactions and cytokine signaling play a crucial role in Bcl6 induction. Bcl6 requirement for T_FH_ development was reported in 2009 by 3 independent groups [24–26]. Indeed, Bcl6 is a master regulator for T_FH_ lineage commitment as its expression can inhibit Th1, Th2, and Th17 differentiation [26]. Bcl6 expression is influenced by IL-6 and IL-21 via STAT1 and STAT3 signaling and by ICOS-PI3K (PhosphoInositide 3-Kinase) signaling. Moreover, Bcl6 expression is controlled by a complex regulatory network of activating factors (see [7] for detailed review) such as basic leucine zipper transcriptional factor ATF like (BATF; [27]), transcription factor 1 (TCF-1; [28]), lymphoid enhancer-binding factor (LEF-1; [28]), and B cell Oct-binding protein 1 (Bob1; [29]), while forkhead box protein O1 (FOXO1; [30]) negatively regulates Bcl6 expression.

### 2.2. Pre-T_FH_ Migration to the T-B Border and T_FH_ Maturation: B Cells Enter the Scene

Thanks to CXCR5 expression enhancement and CCR7 downregulation (Table 2), pre-T_FH_ cells migrate to the B cell follicle in response to a CXCL13 gradient and their interaction with antigen-specific B cells at the T-B border contributes to final T_FH_ differentiation. Indeed, the lower frequency of T_FH_ cells in B cell-deficient mice suggests that B cells are also important for the generation of T_FH_ cells [24]. At this stage, B cells act as the major antigen-presenting cells (APC) for primed-T_FH_ that will then fully differentiate into GC T_FH_ cells. Mature T_FH_ and B cells that have formed stable T-B conjugates move together into the follicle to form GC [31]. Stable T-B conjugate formation requires interaction between ICOS on T_FH_ and ICOSL expressed by B cells, as well as SLAM interactions (Table 2). SLAM are transmembrane receptors expressed on both T_FH_ and B cells. SAP, which is the adaptor signaling protein downstream of SLAM, was demonstrated to be important for stabilizing cognate T-B interactions. Indeed, SAP-deficient CD4^+^ T cells have an impaired capacity to stably interact with cognate B cells, resulting in a failure to induce B cell clonal expansion [32]. Moreover, patients with X-linked Lymphoproliferative disease (XLP), an immunodeficiency resulting from mutations in the SH2D1A gene which encodes SAP, harbor humoral defects characterized by hypogammaglobulinemia and reduced numbers of T_FH_ [33]. B cells thus play a key role in the T_FH_ maturation step by both acting as APC and stabilizing T_FH_-GC B cell interactions through ICOSL and SLAM.

### 2.3. T_FH_ Function: The Final Act of the Story

The major function of T_FH_ is to enhance high affinity memory Ab responses following migration to GC. In the follicles, T_FH_-GC B cell crosstalk involves CD40L, IL-21, PD-1, and BAFF (B cell Activating Factor) (Table 3). The signal delivered through interaction between PD-1 on T_FH_ and PD-L1 expressed by GC B cells is crucial for GC B cell survival [34]. IL-21 production by T_FH_ directly regulates B cell proliferation and class-switch, and the IL-21 pathway has been identified as a critical component of the memory B cell response as secondary antigen-specific IgG responses are impaired in IL-21R-knockout mice [35]. BAFF is a cytokine that belongs to the Tumor Necrosis Factor (TNF) ligand family and its receptors are BCMA (B cell maturation antigen), TACI (Transmembrane Activator and Calcium modulator and Cyclophilin ligand Interactor), and BAFF Receptor 3 (BR3). BAFF is produced by stromal cells in the SLO and involved during GC development by influencing ICOSL expression on B cells and thus regulating the ability of GC B cells to promote T_FH_ expansion [36]. Moreover, BAFF production by T_FH_ is critical for the survival of high affinity B cell clones [37].

In summary, molecules that have been described to play a key role in T_FH_ biology do not display equivalent functions. Some are necessary for T_FH_ migration from the T cell zone to the GC, others are absolutely required for their development or function, and finally some of them are essential for T_FH_ maintenance and survival (Tables 1 [Table tab2]–3).

### 2.4. T_FH_ Regulation

Considering the important role of T_FH_ cells in humoral immunity, a balance between stimulatory and inhibitory mechanisms regulating their function is required for immune homeostasis. However, while signals important for T_FH_ development are clearly defined nowadays, little is known about mechanisms involved in their regulation. The coinhibitory PD-1/PD-L1 pathway can limit T_FH_ expansion and consequently the humoral Ig response [38]. Similarly, it was demonstrated that the inhibitory receptor B and T Lymphocyte Attenuator (BTLA) suppresses GC B cell development and subsequent IgG responses by inhibiting IL-21 production by T_FH_ cells [39] (Table 3). Recently, the existence of regulatory T cells (Treg) able to inhibit GC responses was described. This subset of regulatory T cells of thymic origin was first identified in mice [40] and named T_FR_ (follicular regulatory T cells). They express typical markers of both T_FH_ cells (Bcl6, CXCR5, PD-1, and ICOS) and classical Treg (Foxp3); they localize in the GC and possess suppressive activity. A CD4^+^ T cell population coexpressing Foxp3, Bcl6, and CXCR5 was also visualized in human tonsils [41].

Moreover, microRNA have recently emerged as potent regulators of T_FH_ differentiation. Indeed, the miR-17~92 cluster was shown to promote T_FH_ differentiation by repressing PTEN (Phosphatase and TEnsin homolg), PHLPP2 (Pleckstrin Homology domain and Leucine-rich repeat Protein Phosphatase) (phosphatases that inhibit Bcl6 expression through interfering with PI3K signaling), and ROR*α* (Retinoic acid-related Orphan Receptor *α*) expression [42, 43]. On the other hand, miR-10a negatively regulates T_FH_ differentiation by directly inhibiting Bcl6 expression [44]. Similarly, miR-146a, a microRNA that is highly expressed in T_FH_ cells, was recently described as a negative regulator of T_FH_ cell numbers [45]. miR-146a deficiency leads to accumulation of both T_FH_ and GC B cells, likely due to enhanced ICOSL and ICOS expression on GC B cells and T_FH_ cells, respectively [45].

Finally, IL-2 signaling is also an important negative regulator of T_FH_ differentiation by inducing STAT5-dependent expression of Blimp1, a Bcl6 repressor [46–48]. Moreover, high IL-2 production by Th1 cells induces T-bet, which in turn inhibits Bcl6 expression and T_FH_ differentiation [49].

## 3. Evidences Supporting the Involvement of T_FH_ in Systemic Lupus Erythematosus (SLE)

The main function of T_FH_ cells consists in regulating the clonal selection of GC B cells and providing B cells with signals for Ig production, isotype switching, and somatic hypermutations. As abnormal activation of B cells and autoAb production are central to autoimmune diseases, such as lupus, altered T_FH_ differentiation, function, and regulation were suspected to play a role in lupus pathogenesis. First hypotheses regarding the role of T_FH_ cells in SLE development are based on studies using mice deficient for Roquin1 (a negative regulator of ICOS mRNA stability) in which an excessive number of T_FH_ cells and GC reactions and high levels of IL-21 are associated with a lupus-like phenotype [50, 51]. Other evidences come from studies on IL-21, the main cytokine produced by T_FH_, in lupus mice. High IL-21mRNA as well as elevated IL-21 serum levels were described in BXSB.Y*aa* mice, which develop an SLE-like disease [52]. The use of a fusion protein consisting in the IL-21R linked to the Fc domain of a mouse IgG2a (IL-21R.Fc, which therefore binds to IL-21 and prevents activation of its receptor) revealed a complex biphasic role of IL-21 in this mouse model as it increases or diminishes the disease severity depending of the stage of the disease at the time of IL-21 neutralization (at early or late stages). This could be related to the action of IL-21 on B cells but also on T cell responses [53]. In lupus MRL/*lpr* mice, activated CD4^+^ T cells secrete 10 times more IL-21 than control mice [54] and IL-21R deficiency leads to reduced numbers of T_FH_ cells [55]. In addition, abundant T_FH_-like cells are located outside the GC where they support extrafollicular B cell differentiation and plasmablast maturation in BXSB-Y*aa* and MRL-Fas^lpr^ lupus mice [56, 57]. In the latter and contrary to what was expected, the extrafollicular pathway was shown to be the most important way to generate hypermutated autoAbs [58]. However, there is no evidence to date supporting the involvement of such extrafollicular response in human SLE.

T_FH_ cells are located in SLO; therefore the major problem encountered in studies of human T_FH_ is that lymphoid tissues of lupus patients cannot be easily accessed, making it difficult to identify T_FH_ cells and to determine whether the generation or function of these cells is dysregulated. First studies were based on the enumeration of CD4^+^CXCR5^+^ in peripheral blood as GC T_FH_ counterparts. Using this strategy, it was shown in human SLE that circulating T_FH_ cells (cT_FH_) defined as CD4^+^CXCR5^+^PD-1^+/high^ and/or ICOS^+^ T lymphocytes are expanded in lupus patients and their presence correlates with a more severe disease phenotype [59–64]. Recent studies have more rigorously characterized peripheral CXCR5^+^CD4^+^ T cells. Morita et al. have described a circulating population in healthy donors that shares common phenotypic and functional characteristics with T_FH_ cells from GC [65]. The authors named it T_FH_-like cells. Moreover, they distinguished three subclasses, that is, T_FH_17, T_FH_2, and T_FH_1, defined according to the expression of the CCR6 and CXCR3 chemokine receptors: T_FH_17 cells are CXCR3^−^CCR6^+^ cells whereas T_FH_2 cells are CXCR3^−^CCR6^−^ cells and T_FH_1 cells are CXCR3^+^CCR6^−^ cells. T_FH_17 and T_FH_2 cells were identified as able to provide help to B cells via IL-21 production, resulting in IgM and IgG secretion, whereas T_FH_1 have limited helper functions. However, ICOS expressing T_FH_1 are able to help memory B cells (but not naive B cells) to produce Ab following influenza vaccination [66]. Moreover, Morita and colleagues showed that patients with juvenile dermatomyositis displayed a profound skewing of cT_FH_ cells towards T_FH_2 and T_FH_17 cells that correlated with disease activity, suggesting that an altered balance of T_FH_ subtypes contributes to human autoimmunity [65]. Recently, the differential expression of ICOS, PD-1, and CCR7 interestingly allowed distinguishing three memory cT_FH_ subsets defined as activated cells (ICOS^+^PD1^hi^CCR7^lo^) or quiescent cells (ICOS^−^PD1^+^CCR7^int^ and ICOS^−^PD1^−^CCR7^hi^) [67, 68]. In SLE patients, the frequency of CCR7^lo^PD1^hi^CXCR5^+^ CD4^+^ T cells is significantly higher than in healthy individuals [67]. The CCR7^lo^PD1^hi^ subset is indicative of active T_FH_ differentiation and its overrepresentation is associated with elevated autoAb titers and high disease activity [67]. By analyzing CXCR3 and CCR6 expression, we also interestingly described an altered phenotype of cT_FH_ cells characterized by the enhanced frequency of B cell helper T_FH_2-like CXCR3^−^CCR6^−^ cells and a decreased frequency of CXCR3^+^CCR6^−^T_FH_1-like cells (not able to provide B cell help) in lupus patients with an active disease [69].

## 4. Molecules and/or Cytokines Involved in T_FH_ Generation/Regulation Are Associated with Lupus Pathogenesis

Aberrant expression and/or function of T_FH_-related molecules are associated with lupus-like disease in mice [54, 70]. Similarly, in lupus patients, numbers of molecules involved in T_FH_ generation and/or regulation have been described to be dysregulated.

### 4.1. Surface Molecules

CD40/CD40L pathway plays an essential role in the initial phase of T_FH_ development (T-DC interaction in the T cell zone; Figure 1, [11]) and function (T_FH_-GC B cell crosstalk in the GC; [71]). Interestingly, CD40L was found to be constitutively expressed at abnormally high levels on T cells (but also on B cells and monocytes) from lupus patients [72, 73]. Furthermore, CD4^+^ T cells from female lupus patients, which overexpressed CD40L mRNA, were able to promote autologous B cell stimulation and autoAb production [74].

ICOS-mediated PI3K signaling is absolutely required for T_FH_ differentiation, for T_FH_ migration into the follicle [75], and also for T_FH_ maintenance [76]. PTEN acts as a negative regulator of the PI3K signaling pathway, leading to the inhibition of Bcl6 expression and T_FH_ differentiation. Interestingly, PTEN expression is significantly decreased in SLE B cells [77]; however, to the best of our knowledge, its expression in lupus CD4^+^ T cells (especially T_FH_) has not been investigated yet. ICOS expression has been found to be enhanced in CD4^+^ T cells from lupus patients compared to healthy donors [78, 79] and ICOS levels were higher in patients with nephritis than in those without nephritis [80]. Moreover, infiltrated ICOS^+^ T cells were shown to be in close contact with B cells in lupus kidneys [79].

Interaction between OX40L (on DC) and OX40 (on activated CD4^+^ T cells) is also important for T_FH_ development. OX40 expression by lupus peripheral blood cells was found to be predominantly restricted to memory CD45RO^+^ CD4^+^ T cells and its levels correlated with disease activity [81]. Moreover, OX40 has also been found to be highly expressed in kidneys of patients with lupus nephritis [82]. Importantly, the upstream region of the OX40 gene contains a single risk haplotype for SLE, which is correlated with increased expression of OX40 mRNA and protein [83]. Finally, it was recently shown that OX40 signal promotes,* ex vivo*, the generation of T_FH_-like cells that are functional B cell helpers [84].

### 4.2. Cytokines

Cytokine signals are absolutely required for T_FH_ differentiation. Elevated levels of IL-6 have been found in the serum and in the urine of active SLE patients [85–87]. The increased frequency of IL-6-producing peripheral blood mononuclear cells (PBMC) correlates with disease severity/activity and treatment response [88]. Raised expression of gp130 (one of the two subunits of the IL-6 receptor) has been found on CD4^+^ T cells and B cells from patients with active SLE, while an important reduction in the gp130 expression by B lymphocytes was observed upon immunosuppressive treatment leading to milder disease activity [89]. Factors responsible for the constitutive expression of IL-6 in SLE have not been elucidated yet.

Serum IL-21 levels were found to be elevated in patients with SLE [69, 90], especially in patients with lupus nephritis, and to correlate with disease severity [90]. The real-time PCR analysis of skin biopsies taken from 3 lupus patients also revealed that IL-21 transcripts were significantly increased compared to control individuals [91]. Furthermore, the percentages of CD4^+^ T cells producing IL-21 are significantly enhanced in lupus patients [92]. Finally, polymorphisms within the IL-21R and the IL-21 genes have been reported and may confer risk for SLE: a polymorphism in IL-21R (namely, rs3093301) was found to associate with lupus in 2 independent cohorts [93], a genetic association of two SNPs located in intronic regions of the IL-21 gene (rs2221903 and rs907715) was described [94], and the variant allele rs2055979A of the IL-21 gene was recently found to be associated with increased IL-21 levels [95].

Regarding BAFF, lupus sera have been shown to contain elevated levels of this cytokine and those levels correlate with both anti-dsDNA titers [96–98] and disease activity [99]. Finally, it has been reported that IL-2 production (which inhibits T_FH_ differentiation) upon TCR stimulation is impaired in SLE T lymphocytes [100, 101]. This lower IL-2 production could be explained by imbalanced expression between the transcription factors cAMP response element (CRE) binding protein (CREB) and the CRE-modulator (CREM), which, respectively, enhance and suppress the IL-2 gene transcription [102].

### 4.3. Transcription Factors, miRNA, and Regulatory T Cells

STAT3, which is activated by cytokines such as IL-6 and IL-21, binds to the Bcl6 promoter leading to high levels of Bcl6 expression and is thus important for T_FH_ differentiation. T cells from patients with SLE display increased levels of total and phosphorylated STAT3 [103, 104].

Reduced expression of miR-146a (a negative regulator of T_FH_ development) has been reported in PBMC from SLE patients [105] and seems to correlate with disease activity [105]. Moreover, a genome-wide association study has highlighted a variant, that is, rs2431697, in an intergenic region between PTTG1 (Pituitary Tumor-Transforming 1) and miR-146a, associated with lupus susceptibility [106]. Interestingly, the risk allele of this SNP correlates with a diminution of miR-146a levels [107].

To date, the analysis of frequency and/or functionality of T_FR_ cells in an autoimmune context has not been reported. However, although there may be some discrepancies due to variations in phenotype analysis, peripheral regulatory T cells (CD4^+^CD25^+^ T cells) seem to play a role in human lupus pathogenesis. Several studies reported that a decreased number of Treg might contribute to the pathogenesis [108–111], but there were conflicting data regarding Treg function in lupus patients. The* in vitro* suppressive activity of these cells was found to be defective in some reports [111, 112] but other studies showed that the suppressive activity of highly purified Treg from lupus patients is not altered. It has been proposed that defective suppression in lupus could be attributed either to a higher sensitivity of Treg to Fas-mediated apoptosis in an SLE context [108] or to a lower susceptibility of effector T cells to Treg suppression [113]. Finally, it has been shown that IFN-*α* production by lupus APCs might be responsible for altered Treg functionality [114].

## 5. Targeting T_FH_: From Lupus Mice to Lupus Patients

Data obtained from various lupus mouse models have already highlighted how blockade of signaling pathways involved in T_FH_ generation could lead to disease improvement. The administration of a blocking ICOS-L specific monoclonal Ab (mAb) to lupus NZB/W mice interrupted T_FH_ cell development leading to a decrease of autoAb levels and glomerulonephritis [115, 116]. Similar results were obtained in MRL/lpr lupus mice displaying a genetic deletion of ICOS [57].

Blockade of the CD40L-CD40 signaling pathway also led to the reduction of lupus symptoms in different mouse models [117, 118]. Treatment of MRL/lpr lupus mice with a neutralizing anti-IL-6R mAb has favorable effects on renal function and leads to a reduction of anti-dsDNA Ab levels [119]. In NZB/W mice, chronic administration of anti-IL-6 or anti-IL-6R mAb improves survival and reduces the progression of proteinuria and anti-dsDNA levels [120, 121]. In lupus-prone NZB/W and MRL/lpr mice, raised levels of BAFF are detected at the onset of the disease [122] and treatment with either TACI-Ig or BR3-Ig is effective at preventing clinical disease and ameliorating renal injury [123]. Regarding IL-21, its neutralization using IL-21R.Fc showed an improvement of biological and clinical signs of the disease in MRL/lpr lupus mice and BXSB-*Yaa* mice [53, 54]. Moreover, the administration of Ab specific for the IL-21R to MRL/lpr mice significantly reduced anti-dsDNA Ab titers and IgG deposits in the kidneys when compared to control mice [124]. In NZB/W mice, such IL-21R blocking even allowed reversing nephritis and halting disease progression in mice with preexisting lupus [125]. By using a miRNA-delivery approach via bacteriophage MS2 virus-like particles, Pan and colleagues recently showed that restoring the loss of miR-146a was effective in abolishing autoAb production and delaying SLE progression in lupus-prone mice [126]. Interestingly also, treatment with the small molecule called Stattic (an inhibitor initially reported to block the phosphorylation, dimerization, and nuclear translocation of STAT3 in tumor cells) delayed the onset of proteinuria and reduced both anti-dsDNA autoAb and inflammatory cytokine levels in MRL/lpr lupus mice [127].

**Figure 2:**
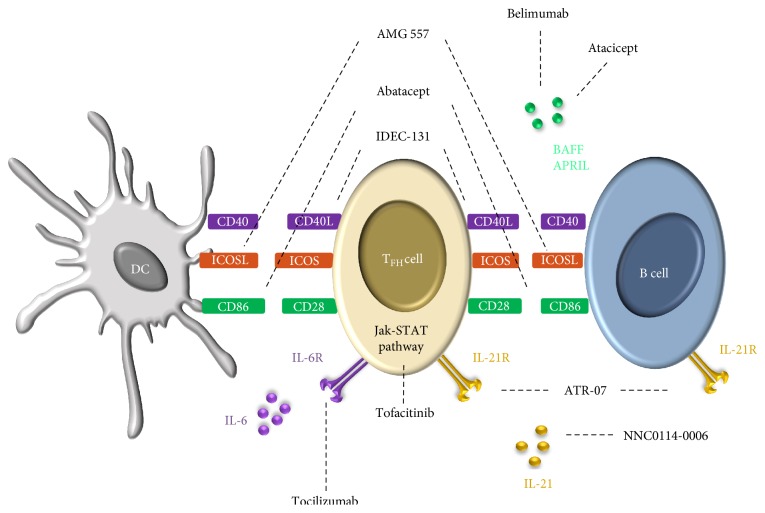
Therapeutic T_FH_-related targets in SLE: present and future. T_FH_ function and differentiation can be affected by several biological drugs already used in SLE therapies or currently in clinical trials. Belimumab, Atacicept, and NNC0114-0006 are mAbs targeting the soluble molecules BAFF, APRIL, and IL-21, respectively. Moreover, the blocking of T cell costimulatory molecules with AMG-557 (ICOSL), Abatacept (CD28), and IDEC-131 (CD40L) could modulate T_FH_ differentiation by decreasing the strength of T-B interactions. Finally, promising therapies could consist in inhibiting T_FH_ differentiation by blocking their signaling pathways either directly with the Jak-STAT inhibitor Tofacitinib or indirectly by the blockade of cytokine receptors such as IL-6R (Tocilizumab) or IL-21R (ATR-07).

There is growing evidence of T_FH_ involvement in the pathogenesis of human SLE. Several therapeutic tools targeting T_FH_ biology already exist and even if their direct effect on T_FH_ development has not been evaluated, some of them were shown to improve the disease. Tocilizumab, a humanized mAb specific for the *α*-chain of the IL-6 receptor (which prevents IL-6 from binding to membrane bound and soluble IL-6 receptors), has been recently tested in SLE patients with promising results [128]. Interestingly, Tocilizumab therapy in rheumatoid arthritis patients leads to a significant reduction in circulating T_FH_ cell numbers and IL-21 production [17]. Belimumab, a human mAb that binds soluble BAFF, therefore inhibiting recognition by BAFF specific receptors has been tested in patients and results from phase III clinical trials have demonstrated the safety profile and efficacy in controlling lupus in a broad range of patients [129]. Belimumab is the first biologic to meet its primary endpoint in a phase III clinical trial for lupus patients and it was approved by the US Food and Drug Administration in 2011. Among other potential therapeutic candidates, are those targeting T-B interactions, such as IDEC-131 (anti-CD40L Ab), AMG 557 (anti-ICOSL Ab), Abatacept (CTLA4-Ig), or targeting cytokines such as ATR-07 (anti-IL-21R Ab), NNC0114-0006 (anti-IL-21 Ab), Atacicept (TACI-Ig), and small molecules inhibiting cytokine signaling pathways (Tofacitinib, a Jak-STAT inhibitor) (Figure 2).

## 6. Concluding Remarks

Although prognosis in SLE has improved markedly in the last 40 years, a better knowledge of the disease remains of prime importance to develop more potent and specific treatments. New targeted therapies designed to block pathways involved in disease pathogenesis are on the horizon. One promising option could be to specifically target factors involved in the generation of plasma cells responsible for the production of pathogenic autoAb in lupus. T_FH_ play a critical role in B cell activation and differentiation, and recent data have evidenced their involvement in lupus pathogenesis. Signals required for T_FH_ development may thus represent interesting targets in order to reduce T_FH_ numbers (and/or to correct the altered proportion of T_FH_ subsets) or to qualitatively and/or quantitatively modulate their function. Another exciting therapeutic option consists in enhancing the negative molecular and cellular regulators of T_FH_, such as miRNA or T_FR_.

## Figures and Tables

**Table 1:** Function of T_FH_-related molecules during T_FH_ differentiation.

T cell molecule	Ligand	Function in mice	Function in humans
CD28	CD80/86	CD28^−/−^ mice fail to form GC [10]	ND

CD40L	CD40	T cell accumulation in B cell follicles relies on CD40-dependent maturation of DC [11]	ND

OX40	OX40L	T cells do not migrate to B cell follicles in immunized OX40^−/−^ mice [11] OX40L instructs CD4^+^ T cells to express CXCR5 [130] CD28^−/−^ T cells fail to upregulate OX40 [10] OX40-OX40L interaction allows CD4^+^ T cells to accumulate in B cell follicles [131]	OX40 signal promotes CD4^+^ T cells to express T_FH_ molecules and to become functional B cell helpers [84]

ICOS	ICOSL	ICOS provides a critical early signal to induce Bcl6 [12] Generation of T_FH_ depends on the PI3K signaling initiated by ICOS [132]	LOF mutations in ICOS reduce cT_FH_ frequencies [133]

IL6R	IL-6	IL-6 promotes the differentiation of naive T cells in helper B cells [14] IL-6^−/−^ mice harbor reduced Bcl6 expression and T_FH_ differentiation [134]	Plasmablasts-derived IL-6 induces T_FH_ differentiation [17]

IL-12R	IL-12	ND	IL-12 induces CD4^+^ T cells to become IL-21-producing T_FH_-like cells [15] IL-12 induces naive CD4^+^ T cells to acquire T_FH_ characteristics and the ability to provide B cell help [16]

IL-21R	IL-21	T cells activated by IL-21 acquire T_FH_ gene expression and function [18] IL-21^−/−^ mice have reduced T_FH_ differentiation and GC formation [134]	LOF mutations in IL-21R skewed T_FH_ differentiation toward an IFN*γ* ^+^PD1^+^ phenotype [133]

GC: germinal center; DC: dendritic cells; ND: not determined; LOF: loss of function; cT_FH_: circulating T_FH_; PC: plasma cells.

**Table 2 tab2:** Function of T_FH_-related molecules during T_FH_ migration and interaction at the T/B border.

T cell molecule	Ligand	Function in mice	Function in humans
CXCR5	CXCL13	CXCR5 induction is necessary for T cell homing to the follicles [135]	T cells localized into B cell follicles express CXCR5 and provide B cell help [136, 137]

CCR7	CCL19/CCL20	Maintenance of CCR7 expression impedes the entry of T cells on the follicles [135]	CXCR5^+^CD4^+^ T cells loose CCR7 expression in SLO [136, 137]

ICOS	ICOSL	CD4^+^ T cells fail to develop in T_FH_ and to promote optimal GC responses when follicular B cells do not express ICOSL [75]	ND

SAP	SLAM	CD4^+^ T cells from SAP^−/−^ mice are unable to stably interact with cognate B cells [32]	XLP patients display reduced T_FH_ numbers and no mem B cells [33]

GC: germinal center; SLO; second lymphoid organs; XLP: X-linked lymphoproliferative disease; ND: not determined; mem B cells: memory B cells.

**Table 3:** Function of T_FH_-related molecules during B cell help, T_FH_ maintenance, and regulation.

T cell molecule	Ligand	Function in mice	Function in humans
CD40L	CD40	The formation of GC and the generation of mem B cells is inhibited in the absence of CD40L [138]	CD40-CD40L interaction is required for the survival of GC B cells [71]

ICOS	ICOSL	T_FH_ are lost in the absence of B cells [12]	Patients with LOF mutation in ICOS have reduced numbers of mem B cells [133]

CD28	CD80	CD80 expression on follicular B cells and its interaction with CD28 on T cells is essential for maintenance of the T_FH_ phenotype [139]	ND

IL-21	IL-21R	IL-21 promotes the differentiation of B cells to mem B cells and PC [52] Mem B cells and PC fail to expand following immunization in IL21^−/−^ mice [35]	B cell differentiation by tonsillar CXCR5^+^ T cells is mediated by IL-21 [140]

IL-4	IL-4R	GC T_FH_ cells produce IL-4, which is required for optimal B cell help [141, 142]	ND

BAFF	BR3/TACI/BCMA	TACI^−/−^ mice have reduced numbers of PC due to a failure in downregulating Bim [36] GC T_FH_ produce BAFF and T-cell restricted BAFF deficiency impairs affinity maturation [37]	ND

PD-1	PD-L1/PD-L2	GC B cell survival is decreased in the absence of PD-1 [143]	CXCR5^+^PD-1^high^ T cells promote antibody responses [67]

BTLA	HVEM	Numbers of IL-21-producing T_FH_-like cells are increased in BTLA^−/−^ mice [39]	ND

SAP	SLAM	IL-4 production by SLAM^−/−^T_FH_ cells is markedly reduced [142]	ND

GC: germinal center; ND: not determined; LOF: loss of function; mem B cells: memory B cells; PC: plasma cells.
